# Antiplatelet therapy for the prevention of atherosclerosis in chronic kidney disease (ALTAS-CKD) patients: study protocol for a randomized clinical trial

**DOI:** 10.1186/s13063-020-04992-x

**Published:** 2021-01-07

**Authors:** Jiachuan Xiong, Ting He, Zhikai Yu, Ke Yang, Feng Chen, Jingbo Cheng, Yu Shi, Yinghui Huang, Yu Qiao, Haiyang Li, Yunzhu Shen, Jinghong Zhao

**Affiliations:** Department of Nephrology, the Key Laboratory for the Prevention and Treatment of Chronic Kidney Disease of Chongqing, Kidney Center of PLA, Xinqiao Hospital, Army Medical University (Third Military Medical University), Chongqing, 400037 People’s Republic of China

**Keywords:** Cardiovascular events, Atherosclerosis, Chronic kidney disease, Antiplatelet, Randomized controlled trial

## Abstract

**Background:**

Cardiovascular disease (CVD) is the most common complication and the leading cause of death in patients with chronic kidney disease (CKD). Accelerated atherosclerosis is a pathophysiological process that is vital to the occurrence of cardiovascular complications associated with CKD. Abnormal platelet activation is not only the leading cause of atherosclerosis but also plays a critical role in the occurrence of thrombotic events. Currently, antiplatelet drugs are commonly used as a secondary prevention strategy for high blood pressure, obesity, diabetes, and ischemic heart disease and can reduce the risk of CVD in the susceptible population. However, the benefits and evidence of using antiplatelet agents in patients with CKD remain controversial. This study aimed to determine whether antiplatelet therapy can safely prevent atherosclerosis in patients with CKD in the primary care setting.

**Methods/design:**

The ALTAS-CKD study is a multicenter, prospective, randomized, double-blind, placebo-controlled trial of 554 adult patients with stage 3–5 non-dialysis-dependent CKD recruited from 10 territory medical centers in China. A secured web-based computer randomization system will be used to administer aspirin 100 mg once daily or a matching inactive placebo for 36 months. The primary endpoint will be the occurrence of atherosclerosis, as measured by carotid ultrasonography. The secondary endpoints will be combined cardiovascular events, all-cause mortality, and 50% decrease in the estimated glomerular filtration rate.

**Trial registration {2a}:**

Current controlled trials number: ChiCTR1900021393. Registered on 18 February 2019.

## Introduction

### Background and rationale {6a}

The prevalence of chronic kidney disease (CKD) in western countries and China is reported to be 11% [1, 2]. Once CKD occurs, it may eventually progress to chronic renal failure and end-stage renal disease (ESRD) [3]. Cardiovascular disease (CVD) is the most common complication and the leading cause of death in patients with CKD and ESRD. The cardiovascular mortality rate in uremia patients ranges from 44 to 51% [4]. Moreover, the risk of cardiovascular mortality in patients with CKD stage 5D aged between 25 and 34 years is 100–150 times higher than that in other age groups [5]. Even in CKD patients aged 60 years and older, the CVD mortality is five times higher than that in the general population (participants were aged 18 years or older and from low- and middle-income countries) [6, 7]. Therefore, it is essential to clarify the critical mechanism underlying the development of cardiovascular complications in patients with CKD and establish an effective treatment.

Previous studies have indicated that CKD-induced atherosclerosis is the primary pathophysiological process underlying the occurrence of cardiovascular complications in patients with CKD. The acceleration of atherosclerosis in CKD patients causes plaque instability [8]. Consequently, arterial plaque rupture can easily result in thromboembolic diseases [9]. Abnormal platelet activation and progression play an essential role in the development of atherosclerosis and trigger the occurrence of physiological hemostasis and various diseases [10, 11]. Previous studies have found that the abnormal activation of platelets is not only a key role in thrombotic events but also the cause of atherosclerosis occurrence and development [12].

The relationship between CKD platelet activation and accelerated atherosclerosis has drawn considerable attention in recent years. Other researchers and our previous studies have found that abnormal platelet activation is manifested by an increase in platelet reactivity in CKD patients and animal models [13–15]. Previous extensive population-based studies have shown that atherosclerosis is highly prevalent in CKD patients, thus increasing the risk of cardiovascular and all-cause mortality. CKD patients have more severe atherosclerosis than age- and sex-matched controls from the general population [6]. The NEFRONA study has indicated that the incidence rates of carotid atherosclerosis are 50.8% in patients with CKD and 69% in diabetic patients with CKD [16]. Currently, antiplatelet drugs are widely used to prevent CVD, especially intravascular thrombosis. Antiplatelet drugs can reduce CVD deaths by 15% and CVD incidence by 20% in high-risk patients [17]. The American Heart Association recommended that antiplatelet drugs can be used as a secondary prevention strategy for patients with high blood pressure, muscular atrophy, obesity, diabetes, or a family history of ischemic heart disease [17]. Although antiplatelet drugs have been shown to reduce the risk of CVD in patients with CKD, the benefits and evidence of using antiplatelet agents in CKD patients remain unknown. A previous meta-analysis showed that antiplatelet drugs could reduce the risk of myocardial infarction in patients with CKD but were not associated with all-cause and cardiovascular mortality [18]. Simultaneously, the SWEDEHEART study demonstrated that dual antiplatelet therapy could reduce the risk of all-cause mortality, stroke, and reinfarction in acute coronary syndrome patients with CKD [19]. Aspirin is widely used and recommended as the first-line antiplatelet drug in the clinical setting. However, a previous meta-analysis indicated that aspirin had no apparent benefits on the primary prevention of CVD in the CKD population [20]. Thus, the efficacy and safety of antiplatelet therapy, like aspirin, in patients with CKD remain controversial. The evidence is not considered robust as previous researches were small sample cohort studies and lacked large clinical randomized controlled (RCT) studies. In addition, CKD patients were excluded or only those with mild CKD (CKD stages 1–2) were recruited in previous extensive RCT studies to assess the efficacy of antiplatelet therapy. Thus, the use of antiplatelet therapy in patients with CKD has fundamental clinical significance. Therefore, a prospective RCT trial must be performed to determine the significance of antiplatelet therapy in CKD patients.

### Objectives {7}

This study aimed to determine the efficacy and safety of aspirin for preventing atherosclerosis in patients with CKD stages 3–5.

- To evaluate the effect of antiplatelet therapy on accelerated atherosclerosis in patients with CKD
- To evaluate whether antiplatelet therapy can reduce cardiovascular events or mortality in patients with CKD
  Moreover, antiplatelet therapy is considered to be safe for patients with CKD.

### Trial design {8}

**Fig. 1:**
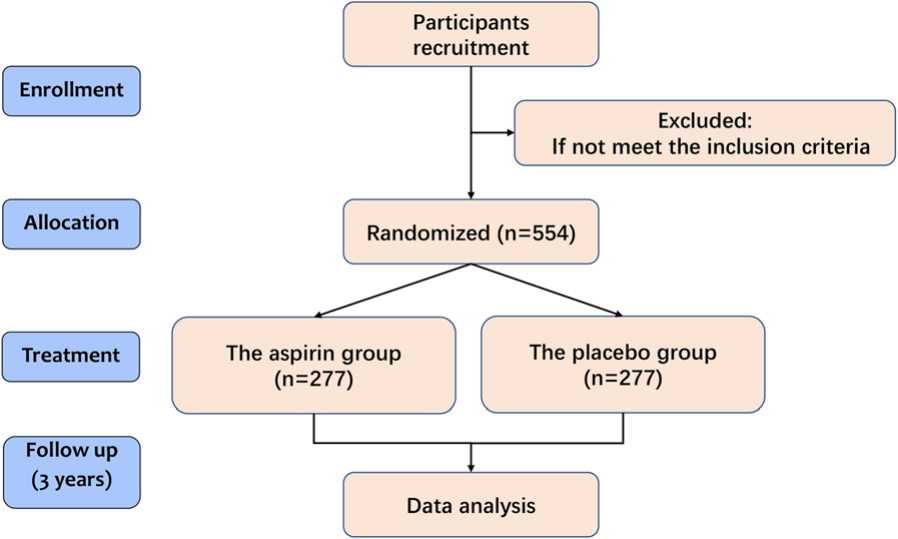
The study design and follow-up

This study is a prospective, open, randomized, controlled multicenter study initiated by the Second Affiliated Hospital of the Army Medical University (Xinqiao Hospital) and approved by the ethics committee of Xinqiao Hospital. An exploratory design will be to investigate the efficacy and safety of aspirin for preventing atherosclerosis in patients with CKD stages 3–5. All eligible patients will be randomly assigned to either the aspirin group or the placebo group (Fig. 1).

## Methods: participants, interventions, and outcomes

### Study setting {9}

The study will be conducted in 10 kidney disease centers in China: Second Affiliated Hospital of Army Military Medical University, Sichuan Provincial People’s Hospital, Tongji Hospital of Tongji University, Union Hospital of Huazhong University of Science and Technology, 2nd Affiliated Hospital of Harbin Medical University, Shanghai East Hospital of Tongji University, Chongqing Chinese Medicine Hospital, Chongqing Bishan District People’s Hospital, Chongqing Qianjiang District People’s Hospital, and Chongqing Qijiang District People’s Hospital.

### Eligibility criteria {10}

#### Participants

##### Inclusion criteria

1. According to the Kidney Disease: Improving Global Outcomes (KDIGO) guidelines, CKD refers to the abnormalities in the kidney structure or function or an estimated glomerular filtration rate (eGFR) of < 60 ml/min/1.73 m^2^ that persists for 3 months with or without kidney damage. The GFR was obtained by calculating the CKD-EPI using the following Cystatin C formula: eGFR=169 × Scr^-0.608^ × CysC^−0.63^ × Age^−0.157^ × (0.83 if female).
2. Patients diagnosed with CKD stages 3–5 (non-dialysis dependent) aged 30–65 years (including those aged 30 and 65 years).
3. Sex and ethnicity restrictions were not imposed.
4. Patients without atherosclerosis in the common carotid artery as observed on the vascular ultrasound examination and no history of atherosclerosis in the carotid artery.
5. An informed consent form from participants is required (Supplementary document).

##### Exclusion criteria

1. Patients who are unable to provide an informed consent
2. Patients who are unable or unwilling to complete the process required by the research
3. Patients who are involved in other interventional clinical trials
4. Pregnant or lactating women
5. Patients with previous cardiovascular diseases such as myocardial infarction, heart failure, and cerebral hemorrhage
6. Patients with NYHA class III or IV heart failure
7. Patients with cirrhosis
8. Patients with HIV infection or AIDS
9. Patients receiving chemotherapy or alkylating agents as treatment for malignant tumors in the past 2 years
10. Kidney transplant patients
11. Patients with deep venous thromboembolism before enrollment
12. Patients using aspirin, clopidogrel, or other antiplatelet drugs before enrollment
13. Patients with active bleeding or coagulopathy dysfunction

##### Who will obtain the informed consent? {26a}

The research investigator at each study location will obtain the informed consent and facilitate participant enrollment. Informed consent from participants will be required. The Patient Information Sheet contains the research background and purpose, research program, study procedures, voluntary principle, rights and responsibilities, expected risks and discomfort associated with the study, treatment measures for research-related damages and expected benefits of the participants, and the consent form explaining the above items in detail.

##### Additional consent provisions for collection and use of participant data and biological specimens {26b}

The collection, processing, and storing of biological specimens will be carried out in accordance with the clinical trial protocol, standard operating procedures, and principles of Good Clinical Laboratory Practice. The storage and use of specimens are described in detail in the patient’s informed consent form. All applicable institutional policies will be followed when handling or discarding specimens after completing all analyses.

### Interventions

#### Explanation for the choice of comparators {6b}

The efficacy and safety of aspirin for the prevention of atherosclerosis in CKD patients have not been proven by large clinical randomized controlled (RCT) studies. In addition, CKD patients were excluded, or only those with mild CKD (CKD stages 1–2) were recruited in previous extensive RCT studies to assess the efficacy of antiplatelet therapy. Thus, the use of antiplatelet therapy in patients with CKD has fundamental clinical significance.

#### Intervention description {11a}

##### Aspirin group

Aspirin regimen: an aspirin dose of 100 mg will be administered daily, and no dietary restrictions will be imposed; if the dose is missed, the medication will be re-administered and taken the next day according to the original plan.

Placebo group: Patients will receive a placebo pill, in which the appearance and shape are consistent with those of aspirin.

##### Consolidated drugs

- High blood pressure should be treated with antihypertensive drugs (target blood pressure < 130/80 mmHg).
- Hypercholesterolemia is primarily treated with a statin, whereas hypertriglyceridemia is primarily treated with fibrate drugs.
- Low-molecular-weight heparin anticoagulants are preferred for anticoagulant treatment.
- Use of the following drugs is prohibited: non-steroidal anti-inflammatory drugs and macrolides such as clarithromycin and erythromycin.
- If the blood sugar level increases during the treatment period, oral hypoglycemic drugs will be administered as initial treatment; if necessary, insulin therapy will be provided as additional treatment.

#### Criteria for modifying or discontinuing intervention {11b}

Participants (1) who cannot tolerate aspirin, (2) in whom treatment was discontinued for more than 2 months due to various reasons, and (3) who will voluntarily withdraw from the study. Those who chose to withdraw from the study will receive appropriate treatment based on the judgment of each central investigator.

#### Strategies to improve adherence to interventions {11c}

Checkups, consultations, and the WeChat app will be used to improve compliance; other methods will also be adopted, such as monitoring and education during monthly appointments. The research team will also remind the participants regarding their upcoming appointment through phone calls or messages sent from the WeChat app.

#### Relevant concomitant care permitted or prohibited during the trial {11d}

During the trial, the participants have access to palliative supportive care for symptom relief, psychological support, and social support. Consolidated drugs could be used under the supervision of the research team. However, any other medical treatments that may influence the trial will be prohibited.

#### Provisions for post-trial care {30}

Post-trial care will not be planned. The possible risks are described in detail in the informed consent form. If any patient suffered harm during the trial, appropriate medical and nursing care will be provided, but not free of charge.

### Outcomes {12}

The primary outcome (see Table 1) is atherosclerosis at treatment endpoint (36 months).

**Table 1:** Primary and secondary outcomes of the study

Outcome	Metric	Time point
**Primary**		
**Atherosclerosis**	Occurrence rate of atherosclerosis	**36** months
**Secondary**		
The combined cardiovascular events	Incidence	**36** months
All-cause mortality	Incidence	**36** months
eGFR decreased by 50%	the proportion of eGFR decreased by 50%	**36** months
Safety outcomes	Numbers	**36** months

Carotid ultrasonography will be used to screen for atherosclerosis. The bilateral carotid arteries will be analyzed by color Doppler ultrasound at a frequency of 10 MHz using a linear array transducer (Model Vinno 70; VINNO Technology (Suzhou) Co., Ltd., China) by an experienced sonographer. A smooth carotid artery is considered normal. An atherosclerotic plaque is defined as a focal structure encroaching into the arterial lumen by at least 0.5 mm or 50% of the surrounding intima-media thickness (IMT) value or with a thickness of > 1.5 mm, as measured from the media-adventitia interface to the intima-lumen interface [21]. Both atherosclerotic plaques and IMT are indicators of carotid atherosclerosis. The carotid ultrasonography will be performed at 6, 12, 24, and 36 months to identify the status of carotid atherosclerosis.

#### Secondary outcomes

1. The combinations of cardiovascular events are as follows:
  - Acute ST-induced myocardial infarction: Evidence of myocardial ischemia, one of the following factors, and the rise or fall of the biochemical marker values (preferably cardiac troponin) with at least one value above the 99th percentile upper reference limit: (A) symptoms of ischemia, (B) new or presumed new significant ST-segment–T wave (ST–T) changes or new left bundle branch block, (C) development of pathological Q waves in the electrogram (ECG), (D) imaging evidence of new loss of viable myocardium or new regional wall motion abnormality, and (E) identification of an intracoronary thrombus on angiography or autopsy.
  - Other acute coronary syndromes: In patients experiencing ischemic symptoms (pain at rest, shortness of breath, press-like discomfort, or progressive exacerbation of the above symptoms), rest or use of nitrates will not relieve these symptoms. On ECG, a transient or persistent ST-segment offset of 0.1 mV or above in one or more leads is usually observed.
  - Congestive heart failure requiring hospitalization: Normal venous reflux is a clinical syndrome characterized by tissue hypoperfusion and pulmonary or systemic circulatory congestion marked by a decrease in cardiac output and an increase in ventricular filling pressure due to the presence of a primary cardiac damage and cardiac insufficiency.
  - Severe arrhythmias: cardiac arrest and resuscitation, ventricular fibrillation, persistent ventricular tachycardia, paroxysmal ventricular tachycardia, primary atrial fibrillation or atrial flutter, severe sinus bradycardia, and complete atrioventricular block after a percutaneous coronary intervention or coronary artery bypass grafting. The occurrence of combined cardiovascular events will be recorded in every visit within the period of 3 years.
2. All-cause mortality. The mortality from any other cause will be registered. These events will be collated during the 3-year follow-up.
3. eGFR decreased by 50%. The eGFR will be calculated at every visit. When the trial is completed, patients in both groups whose eGFR decreased by 50% will be judged as experiencing a renal event.

#### Safety outcomes

1. In the case of severe allergies, the drug should be discontinued immediately, and anti-allergy treatments and symptomatic treatments should be administered immediately.
2. If a severe adverse event occurred, in addition to active treatment or rescue, the time, severity, duration, measures taken, and outcomes of serious adverse events should be recorded.
3. If a treatment-related death occurred, the clinical study should be discontinued immediately, the event should be reported to the ethics committee of the clinical research unit as soon as possible, and the relevant information should be recorded and kept adequately in detail.
4. All adverse events should be monitored until they are adequately resolved or until the patients’ condition stabilizes.

### Participant timeline {13}

The time schedule of enrollment, intervention assessments, and frequency of visits of all participants are shown in a schematic diagram (see the Research flow chart). The participants will be followed up at 0.5, 1, 2, 3, 4, 5, 6, 12, 24, and 36 months.

### Sample size {14}

#### Sample size estimation

The sample was calculated as follows: $n=\frac{{\left({z}_{\alpha}\sqrt{2\overline{pq}}+{z}_{\beta}\sqrt{p1q1+p0q0}\right)}^2}{{\left(p1-p0\right)}^2}$

In this study, p0 and p1 represent the estimated exposure rates of the control group and the experimental group, respectively, while *Z*_α_ and *Z*_β_ indicate the standard positive distribution boundaries. In the table, when *α* or *β* is equal to 0.05, its value is 1.96; when *α* or *β* is equal to 0.01, its value is 2.58. In this study, the incidence of p0 is above 40% [8]. After the intervention, p1 is expected to decrease by 15% compared with p0 (p1 = 25%). Let *α* = 0.05 (both sides), and *β* = 0.10. The calculated minimum sample size is 251; if 10% of the cases were lost to follow-up, the calculated sample size would be 277 for each group, and approximately 554 patients will be included in the trial.

#### Recruitment {15}

Patients from 10 hospital outpatient clinics and inpatients admitted in the department of nephrology will be recruited. The study doctor is responsible for the screening process. The study doctor identifies the potential participants and asks them to participate in the study. Patients will be given sufficient time to consider their participation in the research. Before they decide to participate in this study, discussion with their family and friends is encouraged. If the potential participants have questions regarding the trial, they can ask the research doctor/researcher and will be provided with sufficient explanation until their full understanding is confirmed. To ensure smooth enrollment and shorten enrollment time, all research centers will adopt an effective enrollment strategy to ensure that sufficient study participants are included. We will use the WeChat app (a popular social communication app in China), hospital posters, and oral introduction by the doctor for promotion.

### Methods: assignment of interventions

#### Sequence generation {16a}

Central randomization will be used as a randomization method, and the random number will be generated and assigned by the designated data management center. We will use simple randomization for random allocations. Eligible patients will be randomly assigned to the aspirin group or the placebo group. The treatment will last for 36 months.

#### Concealment mechanism {16b}

To assure concealment, we will use an online central randomization service to randomize the participants. When baseline measurements and the confirmation of eligibility are completed, then the sequence will be revealed.

#### Implementation {16c}

First, the research doctor will complete the baseline measurements and assess if the participants conformed to all the inclusion/exclusion criteria. Finally, the research doctor will use the online central randomization service to assign the participant and complete the assessments and the randomization.

### Assignment of interventions: blinding

#### Who will be blinded {17a}

The study is a double-blind study. The included patients will not be informed about the group to which they belong. The investigators in each center will not know which patients receive active drug or inactive drug.

#### Procedure for unblinding if needed {17b}

If no pregnancy or other emergency occurs during the research period, the blinded participants will be unblinded according to the standard procedure when serious adverse events occurs. If the drugs used in the study have an impact on the treatment, emergency unblinding will be considered.

### Data collection and management

#### Plans for assessment and collection of outcomes {18a}

We will use an electronic data collection form to capture the information using pre-existing forms. Participants will be followed up at 0.5, 1, 2, 3, 4, 5, 6, 12, 24, and 36 months. At each follow-up, routine blood tests, blood biochemistry and coagulation, serum lipid levels, and other related tests will be performed. All biochemical indicators, cervical vascular ultrasound, cardiac ultrasound, and electrocardiography will be performed at 6, 12, 24, and 36 months (see the Research flow chart).

#### Plans to promote participant retention and complete follow-up {18b}

There are no further strategies; those strategies were the same which were described in the improvement adherence to interventions.

#### Collection of basic information

1. Demographic information: age, sex, education, height, weight, CKD stage, body mass index, and comorbidities.
2. Laboratory inspection: including routine blood tests, blood biochemical parameters, and ultrasonic examination.
3. Blood and urine specimens will be obtained at baseline, 1, 6, 12, 24, and 36 months according to the research protocol requirements.
4. Collection of adverse effects and complications: drug-related side effects and complications caused by the disease.

#### Data management {19}

Once the trial is completed, the form will be printed for storage. We will link all data from observations using a unique identification number to facilitate data collection and accuracy.

#### Confidentiality {27}

The collection, storage, and analysis of study data will be carried out in compliance with the relevant data protection regulations during the trial period. Personal information will be replaced with a new identifier, which can only be accessed by the study physician and team member. The research data that need to be taken away from the research center should not contain the personal information of the participants. If necessary, the sponsor, government regulatory authority, or ethics committee may access the patients’ data from the study if required. However, this process will not reveal any information about the participants. After the trial, permission for further storage or use of the remaining specimen should be obtained from the participants, and informed consent will be signed by the participants. Finally, when the trial is finished, the study results will be published in an anonymous form.

#### Plans for collection, laboratory evaluation, and storage of biological specimens for genetic or molecular analysis in this trial/future use {33}

Not applicable for genetic or molecular analysis.

### Statistical analysis

#### Statistical methods for primary and secondary outcomes {20a}

For the primary outcome, the chi-square test will be performed to compare the occurrence rate of atherosclerosis at 1, 2, and 3 years of follow-up between the aspirin group and the placebo group. For the secondary outcome, the chi-square test will be performed to compare the combined cardiovascular events and all-cause mortality at 1, 2, and 3 years of follow-up between the aspirin group and the placebo group. Then, the decrease in eGFR will be computed using the value measured at the last follow-up minus the value measured at baseline; patients with a 50% decline in eGFR will be treated as patients who experienced an eGFR reduction of 50%. Similarly, the proportion of patients with an eGFR reduction of 50% between the aspirin group and the placebo group will be compared using the chi-square test. If the previous comparison did not reach a significant difference, the magnitude of decrease in eGFR between the two groups would be compared using two independent sample *t* tests or the Mann-Whitney *U* tests. In addition, multivariate competing-risk regression models will be used to assess the independent effect of treatments adjusted for age, sex, diabetes, serum lipid profiles, and phosphorus.

All statistical analyses will be performed using the SPSS software version 21.0 (IBM Corp., Armonk, NY, USA), and general data will be expressed as mean ± standard deviation. The variables with normal distribution and variance will be compared. One-way analysis of variance will be used for comparisons within groups. The SNK test will be used for multiple comparisons between groups. Pearson’s correlation analysis will be used for analyzing the correlation between variables. A nonparametric test will be used to analyze variables that do not meet the parameter test conditions. The Kruskal-Wallis *H* test or Mann-Whitney *U* test will be used for data comparison. Spearman’s correlation analysis will be used for correlation analysis. The chi-square test will be used for comparison. A *p* value of < 0.05 will be considered significant. A nonparametric test for multiple comparisons will be corrected using the Bonferroni method, *p* < 0.008, statistically significant safety analysis.

#### Interim analyses {21b}

Not applicable.

#### Methods for additional analyses (e.g., subgroup analyses) {20b}

The efficacy analysis will be based on the intention-to-treat analysis, which will be conducted in all patients who have been randomized and used drugs. There will be no pre-defined subgroup analysis. If unusual or unexpected results occur, then post-hoc subgroup analyses may be used.

#### Methods in analysis to handle protocol non-adherence and any statistical methods to handle missing data {20c}

For missing data imputation, the last observation carried forward method will be used if the missing data met the following hypothesis: missing completely at random.

### Oversight and monitoring

#### Composition of the coordinating center and trial steering committee {5d}

Not available.

#### Composition of the data monitoring committee, its role, and reporting structure {21a}

##### Data and safety monitoring board

An independent Data Safety Monitoring Board (DSMB) has been established to maintain the oversight of patient safety and data integrity within the trial. The DSMB consisted of six members: three expert nephrologists (1 of them assigned as the head nephrologist), an ethicist, a biostatistician, and a patient representative. The DSMB has no financial, proprietary, professional, or other interests that may affect the impartial and independent decision-making in the trial. The DSMB will monitor the trial data. The safety, effectiveness, and integrity of the trial will be ensured.

##### Description of any interim analyses and stopping guidelines {21b}

The DSMB will evaluate the progress of participant recruitment and make recommendations to the sponsor to continue, modify, or terminate or suspend the trial in one or all groups as originally planned. The committee will meet in person or conduct an online conference prior to the trial commencement and then 3 and 6 months after the initiation of the trial. The DSMB will participate in the blinded interim analyses.

##### Adverse event reporting and harms {22}

During the entire treatment and follow-up process, close attention should be paid to observing the adverse events or unanticipated toxic side effects (including symptoms, signs, and laboratory tests). Once an adverse event occurs, regardless of whether the event has a causal relationship with the drug, it should be addressed immediately. Moreover, the investigators should decide whether to terminate the study and conduct a safe visit based on the condition.

##### Frequency and plans for auditing trial conduct {23}

No routine plans for independent trial auditing.

##### Plans for communicating important protocol amendments to relevant parties (e.g., trial participants and ethical committees) {25}

Generally, modifications to the protocol are not commonly performed. However, in a scenario where revisions are made, the research group will submit the revised protocol to the ethics committee if any significant change was made. The ethics committee will review or quickly review the new version of the protocol according to the revised content. Then, the revised protocol will be emailed to the principal investigator in each center.

##### Dissemination plan {31a}

After the trial is completed, the results of the current study will be disseminated to the trial participants, and dissemination of these results to the general public will be made through newsletters and our hospital website. Dissemination to clinicians and experts will be made through the presentation at relevant national and international conferences and publications in an open-access format in peer-reviewed journals.

##### Plans to give access to the full protocol, participant-level data, and statistical code {31c}

Not available.

## Discussion

Previous epidemic studies reported that the incidence rates of atherosclerosis in non-diabetic and diabetic CKD patients are 50% and 69%, respectively [16, 22]. The incidence of atherosclerosis in non-diabetic CKD patients was also proven by conducting an imaging analysis in a Korean study, which enrolled more than 3000 individuals without known CAD who underwent coronary computed tomography angiography. This study showed that 34.1% and 48.6% of the individuals with mild (eGFR 60–89 mL/min/1.73 m^2^) and moderate (eGFR 30–59 mL/min/1.73 m^2^) CKD had segment vessel stenosis and stenosis of ≥ 50%, respectively [23]. Aspirin has been shown to improve atherosclerosis in animal models. Aspirin-triggered lipoxins A4 (ATL4) is an effective anti-inflammatory mediator involved in inflammation regression. ATL4 reduced the macrophage infiltration and apoptosis in ApoE−/− mice with atherosclerosis in the aortic roots and thoracic aorta [24]. This indicates that aspirin can inhibit the expression of nuclear factor-kappa B1 and deactivate the cAMP signaling pathway to treat atherosclerosis [25]. Inflammation plays a vital role in the pathophysiology of atherosclerosis, and aspirin has significant anti-inflammatory properties. In Chai et al.’s study, orchidectomized Sprague-Dawley rats fed with a AIN-93 M casein-based control diet for 4 months developed atherosclerotic lesions. After treatment with aspirin (500 mg/kg) for 3 months, the fatty streak area was completely reversed, and the atherosclerotic lesions disappeared [26]. Therefore, the theoretical mechanism and arterial test of aspirin support its role in improving atherosclerosis.

However, the results of population-based studies remain controversial and inconclusive. Some studies suggested that aspirin can improve atherosclerosis, while other studies showed no difference compared with the placebo control group. Bavry et al. found that the use of aspirin could reduce the occurrence of endpoint events (cardiovascular death, myocardial infarction, or stroke) in the group with previous ischemic events; in the group without previous ischemic events, aspirin did not improve the occurrence of endpoint events [27]. Another study conducted by Ogawa et al. showed that treatment with low-dose aspirin did not prevent atherosclerotic events in patients with type 2 diabetes (including those with ischemic heart disease, stroke, and peripheral arterial disease) [28]. Only 100 patients were included, and the number of endpoint events in the aspirin group appeared to be lower but not statistically significant. A previous meta-analysis involving 10,117 patients suggested that there was no significant difference between the aspirin group and the blank control group in the reduction of all-cause mortality and atherosclerotic events [29]. However, in the CKD population, some studies suggested that low-dose aspirin (100 mg/day) can even increase the occurrence of CVD and CKD progression [30]. Therefore, the protective effect of aspirin in patients with CKD has not been determined.

In summary, if antiplatelet therapy is proven to be effective and safe for the prevention of atherosclerosis in the CKD population managed in a primary care setting in this pilot study, it will undoubtedly facilitate the development of a future definitive large RCT to determine the effect of antiplatelet therapy on hard, decisive endpoints (such as cardiovascular morbidity and mortality).

### Trial status

The trial is currently in the pre-recruitment phase (Supplementary Table 1). The first participant will be hopefully assigned on 1 March 2020. The recruitment will be completed by March 2020. This protocol is version 1.2, dated 18 January 2020. Trial completion will be expected by March 2023. In addition, any protocol amendments will be submitted to the DSMB for review.

## Supplementary Information

**Additional file 1.**

**Additional file 2.**

## Abbreviations

CVD: Cardiovascular disease
CKD: Chronic kidney disease
ESRD: End-stage renal disease
LVH: Left ventricular hypertrophy
CAD: Coronary artery disease
PMP: Platelet microparticle
PMA: Platelet-monocyte aggregates
AHA: American Heart Association
RCT: Randomized controlled trial
ATL4: Aspirin-triggered lipoxins A4
DSMB: Data and Safety Monitoring Board
