# Supplementary material for: Antiplatelet therapy for the prevention of atherosclerosis in chronic kidney disease (ALTAS-CKD) patients: study protocol for a randomized clinical trial
Source: Trials. 2021 Jan 7;22:37. doi: 10.1186/s13063-020-04992-x (PMC7792066; doi:10.1186/s13063-020-04992-x)
Supplement: Supplementary file 1 — Additional file 1. [file 13063_2020_4992_MOESM1_ESM.docx]

| **Supplementary Table 1. The trial registration data set** | |
| --- | --- |
| **Data category** | **Information** |
| Primary registry and trial identifying number | ChiCTR1900021393 |
| Date of registration in primary registry | 2019-02-18 |
| Source(s) of monetary or material support | The training program for clinical medical research of the Army Military Medical University |
| Primary sponsor | The Second Affiliated Hospital of the Amy Medical University |
| Contact for scientific queries | Jinghong Zhao, Email: zhaojh@tmmu.edu.cn |
| Official title | Antiplatelet therapy for prevention of atherosclerosis in chronic kidney disease: A perspective, multi-center randomized controlled trial |
| Countries of recruitment | China |
| Health condition(s) or problem(s) studied | Chronic kidney diseases |
| Intervention(s) | Aspirin (100mg) versus placebo in chronic kidney diseases |
| Key inclusion and exclusion criteria | Inclusion criteria:  (1) Patients diagnosed with CKD stage 3-5, aged 30-65 years.  (2) Sex and ethnicity are not limited.  (3) There was no atherosclerosis in the common carotid artery through the vascular ultrasound examination, and no atherosclerosis of the carotid artery was reported previously.  (4) An informed consent form from participants was required (supplementary document).  Exclusion criteria:  (1) Unable to provide informed consent.  (2) Unable or unwilling to complete the process required by the research.  (3) Being involved in other interventional clinical trials.  (4) Pregnant or lactating women.  (5) Patients with previous cardiovascular diseases such as myocardial infarction, heart failure, and cerebral hemorrhage.  (6) NYHA class III or IV heart failure.  (7) Cirrhosis.  (8) HIV infection or AIDS.  (9) Receiving chemotherapy or alkylating agents for malignant tumors in the past two years.  (10) Kidney transplant patients.  (11) Deep venous thromboembolism before enrollment.  (12) Aspirin, clopidogrel, or other antiplatelet drugs were currently used before enrollment.  (13) Patients with active bleeding or coagulopathy dysfunction. |
| Study type | Interventional study |
| Date of first enrolment | 1 March 2020 |
| Estimated primary completion date | 1 March 2023 |
| Protocol version number and date | version 1.2, 18 January 2020 |
| Recruitment status | Ongoing |
| Primary outcome(s) | Atherosclerosis |
| Key secondary outcomes | (1) The combined cardiovascular event; (2) All-cause mortality; (3) eGFR decreased by 50%; (4) Safety outcomes. |
