# Supplementary material for: Antiplatelet therapy for the prevention of atherosclerosis in chronic kidney disease (ALTAS-CKD) patients: study protocol for a randomized clinical trial
Source: Trials. 2021 Jan 7;22:37. doi: 10.1186/s13063-020-04992-x (PMC7792066; doi:10.1186/s13063-020-04992-x)
Supplement: Supplementary file 2 — Additional file 2. [file 13063_2020_4992_MOESM2_ESM.docx]

|  | **Research flow chart** | | | | | | | |
| --- | --- | --- | --- | --- | --- | --- | --- | --- |
| **Items** | The baseline visit within 72 hours after randomization | Second visit  2 weeks (3 days) | Third visit  1 month (1 week) | The fourth visit 3 months (1 week) | The fifth visit 6 months (1 week) | The sixth visit 12 months (1 week) | Seventh visit  24 months (1 week) | The eighth visit  36 months (1 week) |
| Inclusion/exclusion criteria check | ☑ |  |  |  |  |  |  |  |
| Subject information and informed consent | ☑ |  |  |  |  |  |  |  |
| Demographic information | ☑ |  |  |  |  |  |  |  |
| History of present illness and concomitant diseases | ☑ | ☑ | ☑ | ☑ | ☑ | ☑ | ☑ | ☑ |
| Physical check | ☑ | ☑ | ☑ | ☑ | ☑ | ☑ | ☑ | ☑ |
| Blood routine | ☑ | ☑ | ☑ | ☑ | ☑ | ☑ | ☑ | ☑ |
| Liver function test | ☑ | ☑ | ☑ | ☑ | ☑ | ☑ | ☑ | ☑ |
| Renal function test | ☑ | ☑ | ☑ | ☑ | ☑ | ☑ | ☑ | ☑ |
| Coagulation function test | ☑ |  |  |  | ☑ | ☑ | ☑ | ☑ |
| D-dimer | ☑ |  |  |  | ☑ | ☑ | ☑ | ☑ |
| Myocardial enzyme spectrum | ☑ |  |  |  | ☑ | ☑ | ☑ | ☑ |
| Blood fat | ☑ | ☑ | ☑ | ☑ | ☑ | ☑ | ☑ | ☑ |
| Blood sugar | ☑ | ☑ | ☑ | ☑ | ☑ | ☑ | ☑ | ☑ |
| CRP | ☑ |  |  |  |  |  |  |  |
| Electrocardiogram (ECG) | ☑ |  |  |  | ☑ | ☑ | ☑ | ☑ |
| Neck blood vessel color doppler ultrasound | ☑ |  |  |  | ☑ | ☑ | ☑ | ☑ |
| Echocardiography | ☑ |  |  |  | ☑ | ☑ | ☑ | ☑ |
| pregnancy | ☑ |  |  |  |  |  |  |  |
| randomization | ☑ |  |  |  |  |  |  |  |
| Distribution of research drugs | ☑ | ☑ | ☑ | ☑ | ☑ | ☑ | ☑ | ☑ |
| Medication record |  | ☑ | ☑ | ☑ | ☑ | ☑ | ☑ | ☑ |
| Take back any excess research drugs |  | ☑ | ☑ | ☑ | ☑ | ☑ | ☑ | ☑ |
| Along with the medicine | ☑ | ☑ | ☑ | ☑ | ☑ | ☑ | ☑ | ☑ |
| Bleeding events |  | ☑ | ☑ | ☑ | ☑ | ☑ | ☑ | ☑ |
| Other security events |  | ☑ | ☑ | ☑ | ☑ | ☑ | ☑ | ☑ |
| Thromboembolic events |  | ☑ | ☑ | ☑ | ☑ | ☑ | ☑ | ☑ |
| Death |  | ☑ | ☑ | ☑ | ☑ | ☑ | ☑ | ☑ |
| Research completed |  |  |  |  |  |  |  | ☑ |
